# Extraction of Nicotine from Tobacco Leaves and Development of Fast Dissolving Nicotine Extract Film

**DOI:** 10.3390/membranes11060403

**Published:** 2021-05-28

**Authors:** Kantaporn Kheawfu, Adchareeya Kaewpinta, Wisinee Chanmahasathien, Pornchai Rachtanapun, Pensak Jantrawut

**Affiliations:** 1 Department of Pharmaceutical Sciences, Faculty of Pharmacy, Chiang Mai University, Chiang Mai 50200, Thailand; kantaporn.kheawfu@cmu.ac.th (K.K.); wisinee.c@cmu.ac.th (W.C.); 2 Research Center of Pharmaceutical Nanotechnology, Faculty of Pharmacy, Chiang Mai University, Chiang Mai 50200, Thailand; 3 Interdisciplinary Program in Nanoscience and Nanotechnology, Faculty of Science, Chiang Mai University, Chiang Mai 50200, Thailand; adchareeya_k@cmu.ac.th; 4 Division of Packaging Technology, School of Agro-Industry, Faculty of Agro-Industry, Chiang Mai University, Chiang Mai 50100, Thailand; pornchai.r@cmu.ac.th; 5 Cluster of Agro Bio-Circular-Green Industry (Agro BCG), Chiang Mai University, Chiang Mai 50100, Thailand

**Keywords:** nicotine, tobacco, extraction, fast dissolving formulation, thin film

## Abstract

Nicotine (NCT), administered in the form of a fast dissolving oral delivery system, can be a potential alternative to nicotine replacement therapy. NCT was extracted by maceration and acid-base extraction methods from Burley tobacco leaves with different stalk positions and extraction yield and NCT content were further determined. The extract with the highest nicotine content was selected for incorporation into a fast dissolving film formulation. The optimized film was evaluated for its physical and mechanical properties, *in vitro* disintegration, and drug release profile. The results demonstrated that the extract from the upper part of tobacco leaves using the acid-base extraction method had the highest amount of NCT. NCT fast dissolving film consisting of this extract as the active ingredient and HPMC E15 as a film polymer resulted in a homogeneous translucent film with a light brown color. The addition of NCT significantly affected the film properties in terms of weight, disintegration time, tensile strength, percentage elongation at break, and Young’s modulus values. The drug release of NCT fast dissolving film showed a rapid initial release of 80% within three minutes, and its kinetics followed the Higuchi matrix model. The results suggest that these NCT films can be employed in the development of NCT fast dissolving films for clinical use.

## 1. Introduction

**Figure 1:**
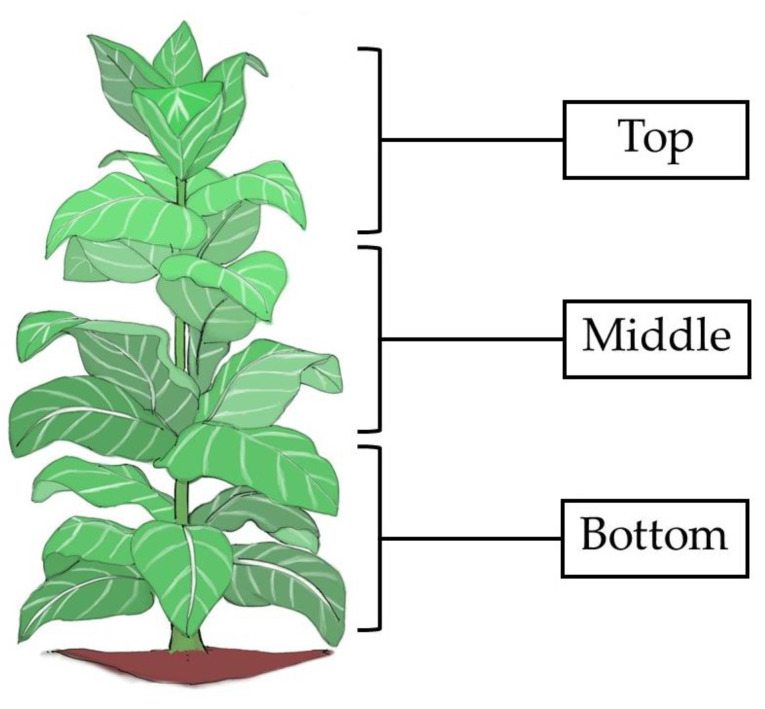
Burley tobacco plant variety showing approximate stalk position of farmers’ grades.

Tobacco (*Nicotiana tabacum* L.) belongs to the genus Nicotiana and is native to America. This plant is farmed over the northern and northeastern area in Thailand and has utility primarily for the raw material for making cigarettes under the Tobacco Authority of Thailand (TOAT) organization. Unlike most crops, it is the leaves of the tobacco plant that are of economic importance. Beginning at the bottom of the plant, tobacco leaves are generally classified into four main groups: lug, cutter, leaf, and tip [1]. The lowermost four or five leaves, which are referred to as lugs, have the lowest nicotine concentration, have the highest reducing sugar concentration, and contain the least amount of aroma and flavor relative to the middle (cutter) and upper-stalk (leaf and tip) positions [1]. Burley tobacco, which is an important economic tobacco variety in Thailand, is marketed on a grade basis, and the position of the leaves on the stalk is closely related to the grade. Therefore, the chemical composition of the leaves as related to position on the stalk should supply valuable information regarding their suitability for a particular use. Nicotine (NCT) is an alkaloid contained in tobacco leaves. NCT is soluble in some types of solvents, such as alcohol, chloroform, ether, petroleum ether, kerosene, and water. Therefore, various solvents can be used to isolate nicotine from tobacco leaves by using a solvent extraction method [2]. NCT is widely used in fine chemical, pharmaceutical, and agricultural industries as well as in the tobacco industry itself as an essential cigarette additive. As a pharmaceutical drug, NCT is used for smoking cessation to relieve withdrawal symptoms [3]. NRT via oral administration is limited due to the rapid destruction of NCT from first-pass metabolism [4]. Currently, many dosage forms of NCT have been developed, e.g., transdermal patches, mouth sprays, lozenges, chewing gum, oral films, etc. Each of these dosage forms has advantages and disadvantages. A transdermal patch is a slow, sustained-release form different from cigarette smoking. The most common side effect is a local skin reaction, which occurs in the contact area and requires changing patch sites daily [5]. Despite the permeability of skin and buccal mucosa, all oral regions are more permeable than skin because the human buccal mucosa is composed of non-keratinized cells [6]. Mouth sprays can increase the absorption of NCT. This dosage form needs to be administered often since the NCT does not persist in the bloodstream for a long time. The instructions on using NCT gum are quite complicated, as patients need to slowly chew the NCT gum until they can taste the NCT, at which point they must stop chewing and hold the NCT gum between the cheek and gums for about a minute, then resume chewing, repeating this process for about 30 min [7]. This chewing and swallowing behavior affects the variable onset and blood levels of nicotine [8]. Therefore, NCT administered in the form of a fast dissolving oral delivery system may reduce variability from chewing or swallowing behavior and has the potential to become a regular technique in combating withdrawal symptoms in smokers who are in the process of smoking cessation. Fast dissolving oral delivery systems are solid dosage forms that disintegrate or dissolve rapidly (less than 1 min) when placed in the mouth, without drinking or chewing [9]. Although this dosage form requires frequent drug administration, it is an easily portable form that improves patient convenience and medication adherence. In the development of these dosage forms, the main critical issues are represented by dissolution in the oral cavity and the tensile properties required for packaging. The concept of fast dissolving drug delivery systems emerged from the desire to provide patients with an easy way of taking their medication. The delivery system consists of a very thin oral strip, which is simply placed on the patient’s tongue or any oral mucosal tissue. Instantly wet by saliva, the film rapidly hydrates and adheres onto the site of application [10]. It then rapidly disintegrates and dissolves to release the medication for oromucosal absorption [11]. Thus, the present investigation aimed to extract NCT from different positions (lower, middle, and upper) of tobacco leaves (Figure 1) by maceration and acid-base extraction methods and determine the NCT content in the extracts. The extract with the highest nicotine content was selected to incorporate into fast dissolving film formulations. The NCT fast dissolving film was evaluated for physical and mechanical properties as well as *in vitro* disintegration and dissolution.

## 2. Materials and Methods

### 2.1. Plant Materials and Chemicals

The light air-cured leaves of *N. tabaccum* were received from Maejo Tobacco Experiment Station, Chiang Mai, Thailand, under the TOAT organization. The plant voucher specimen (No. 0023267) was deposited at the Herbarium of the Faculty of Pharmacy, Chiang Mai University (CMU), and authenticated by the CMU staff botanist. Chloroform, ethanol, glacial acetic acid, methanol, and sodium acetate were purchased from RCI Labscan Limited (Bangkok, Thailand). Sodium carbonate and sodium hydroxide were purchased from Merck (Darmstadt, Germany). Ethanol (99% *v*/*v*) was purchased from Liquor Distillery Organization (Bangkok, Thailand). Trimethylamine was purchased from Loba Chemie Pvt, Ltd. (Mumbai, India). Nicotine standard was purchased from Sigma-Aldrich (St. Louis, MO, USA). Hydroxypropyl methylcellulose E15 (HPMC E15, AnyCoat^®^-C AN15) was purchased from Lotte Fine Chemical Co., Ltd. (Seoul, Korea).

### 2.2. Preparation of Nicotine (NCT) Extract

#### 2.2.1. Extraction of Tobacco Leaves

The three parts of *N. tabaccum* leaves—top, middle, and bottom—were dried in an oven at 55 °C for 2 h and ground to a powder. The dry *N. tabaccum* leaves were extracted by 3 methods: water maceration extraction, ethanol maceration extraction, and acid-base extraction. For maceration extraction, 50 g of tobacco leaves were macerated (48 h × 3 times) at room temperature (25 ± 2 °C) in 750 mL of each solvent. The extracts from the 3 macerations were pooled, filtered and subjected to a rotary evaporation to obtain crude extracts. Acid-base extraction is based on the alkaloid property of nicotine, which involves different solubility levels in water and an organic solvent. The 50 g of tobacco leaves was boiled with 750 mL of water at 80 ± 5 °C for 20 min, then 10 g of sodium carbonate was added and the mixture was continuously heated for 10 min. After filtration, the obtained filtrate was adjusted to pH 12 using sodium hydroxide and extracted with chloroform (100 mL × 2 times) using the liquid-liquid extraction technique. The chloroform of the filtrate was removed using a rotary evaporator at 50 °C under vacuum to obtain crude extracts [12]. Each extraction method was performed in duplicate. The obtained crude extracts were stored at 4 °C and protected from light until further study. The calculation of tobacco leaf extract yield is shown in Equation (1), as follows:

(1) $$\mathrm{Yield}\;\mathrm{of}\;\mathrm{tobacco}\;\mathrm{leaves}\;\mathrm{extract}=\frac{\mathrm{Weight}\;\mathrm{of}\;\mathrm{extract}}{\mathrm{Dry}\;\mathrm{weight}\;\mathrm{of}\;\mathrm{tobacco}\;\mathrm{leaves}}\times 100\%$$

#### 2.2.2. Determination of NCT Content in the Extracts

NCT yield percentage was analyzed by HPLC using an Agilent HP1100 HPLC instrument (Agilent Technologies, Santa Clara, CA, USA) with an autosampler. The stationary phase used was a C-18 reverse-phase column, 4.6 × 150 mm (Agilent technologies, Santa Clara, CA, USA). Sodium acetate solution, methanol, and trimethylamine, (88:12:0.5 *v*/*v*) were used as mobile phase, with the pH adjusted to 4.2 using glacial acetic acid, at a flow rate of 1 mL/min and UV detection at 259 nm [13]. The retention time of NCT was detected at approximately 3.2 min. A calibration curve was plotted from NCT standards ranging from 5 µg/mL to 250 µg/mL in water (R^2^ = 0.9999) and ethanol (R^2^ = 0.9997). The calculation of NCT per tobacco leaf extract yield is shown in Equation (2), as follows:

(2) $$\mathrm{Yield}\;\mathrm{of}\;\mathrm{NCT}\;\mathrm{content}\;\mathrm{in}\;\mathrm{the}\;\mathrm{extract}=\frac{\mathrm{Weight}\;\mathrm{of}\;\mathrm{NCT}}{\mathrm{Weight}\;\mathrm{of}\;\mathrm{tobacco}\;\mathrm{leaves}\;\mathrm{extract}}\times 100\%$$

### 2.3. Preparation and Characterization of NCT Fast Dissolving Films

#### 2.3.1. NCT Fast Dissolving Film Preparation

The NCT fast dissolving films were fabricated using the solvent casting technique. A hydroalcoholic solution (ethanol:water = 9:1) of 5% *w*/*w* HPMC E15 was prepared by dispersing HPMC E15 in distilled water and stirring at room temperature (25 ± 2 °C) for 30 min. Then, 0.51% *w*/*w* NCT extract was added to the HPMC E15 solution, gently stirred for 5 min, and left until all appearing air bubbles had disappeared. Ten grams of the prepared solution was cast onto a Petri dish with a 9 cm diameter (the area of the Petri dish was 63.64 cm^2^) and then dried at room temperature overnight. The obtained film was cut into a square shape with a size of 2 cm × 2 cm, containing approximately 2 mg of NCT. Blank films without NCT were prepared and characterized to compare with NCT fast dissolving films.

#### 2.3.2. Characterization of NCT Fast Dissolving Films

##### Morphology Characterization of NCT Fast Dissolving Films

The morphology of NCT fast dissolving films was investigated by scanning electron microscopy (SEM, JEOL JSM-6610LV, Tokyo, Japan). A square-shaped film with a size of 5 mm × 5 mm was cut to investigate film surface, whereas a rectangle-shaped film with a size of approximately 1 mm × 5 mm was cut to investigate film thickness. The film was placed on a carbon tape, and the film surface was then coated with gold for 15 s using a 40 mA sputter coater (JEOL JFC-1100E, Tokyo, Japan). The SEM images were taken at an accelerating voltage of 15 kV with 350× and 500× magnifications.

##### Film Thickness

The thickness of each 2 cm × 2 cm piece of film was measured at three points (left, middle, and right) in the same position on each film by an outside micrometer (3203-25A, Insize CO, Ltd., Suzhou New District, Jiangsu, China). Five replicates were conducted for each film, and the average film thickness (in mm) with standard deviation was calculated.

##### Film Weight

Three pieces of 2 × 2 cm^2^ size were cut randomly from each film formulation. Films were weighed individually on an electronic balance (PA214, Ohaus Corporation, Parsippany, NJ, USA), and the mean weight was calculated.

##### *In Vitro* Disintegration Time Study

The disintegration test method used in this study was modified from Preis et al. (2014) [14]. The film was clamped between the sample holder and the magnetic clip (attached to the bottom side of the film). The magnetic clip had a weight of 3 g (0.03 N), which represented the approximate minimal force applied by the human tongue. Then, the attached film was half-immersed (50%) in 65 mL of simulated salivary fluid with pH 6.8 at 37 ± 0.5 °C. The simulated salivary fluid was composed of sodium chloride 8.0 g/L, potassium phosphate monobasic 0.19 g/L, and sodium phosphate dibasic dihydrate 2.98 g/L. The pH was adjusted to 6.8 with 1 M hydrochloric acid [15]. The time required for the film to break and the magnetic clip to drop down was recorded visually and noted as *in vitro* disintegration time. All studies were performed in triplicate for each formulation. For the direct comparisons of *in vitro* disintegration times, the obtained disintegration times were normalized by thickness of each film.

##### Mechanical Strength Test

Mechanical strength of the films was tested using a texture analyzer TX.TA plus (Stable Micro Systems, Surrey, UK). An individual sample holder was constructed to facilitate measurements of 2 cm × 2 cm sized film samples. The dry film was fixed on the plate with a cylindrical hole with a 9.0 mm diameter (area of the sample holder hole = 63.56 mm^2^). A cylindrical stainless probe (2 mm diameter) with a plane flat-faced surface was used (probe contact area = 3.14 mm^2^). The texture analyzer was adjusted for the probe’s forward movement at a velocity of 1.0 mm/s. Measurement started when the probe had contacted the sample surface (triggering force). The probe moved on at constant speed until the film was torn. The applied force and distance were recorded. All of the experiments were conducted at room temperature (25 ± 2 °C, 70% relative humidity). Five replicates were conducted for each film. The mechanical strength of the film was characterized by tensile strength, elongation at break, and Young’s modulus [16,17,18].

### 2.4. NCT Loading Efficiency

Three randomly taken films (size of 2 cm × 2 cm) were added into vials containing 10 mL of 50% *v*/*v* ethanol and gently stirred for 15 min or until the film dissolved completely. The solution was withdrawn, filtered, diluted 2 times with 50% *v*/*v* ethanol, and then analyzed by HPLC, following the method specified in Section 2.2.2. NCT contents were determined from the standard curve of NCT in 50% *v*/*v* ethanol, which demonstrated linearity with a high correlation coefficient (R^2^ = 0.9992). The following regression equation was obtained: *y* = 5.0003 *x* + 7.9187, where *y* is the absorbance and *x* is the concentration of NCT (µg/mL). The experiment was done in triplicate. The percentage of NCT loading efficiency was calculated using Equation (3), as follows:

(3) $$\mathrm{NCT}\;\mathrm{loading}\;\mathrm{efficiency}\;\left(\%\right)=\frac{\mathrm{Mass}\;\mathrm{of}\;\mathrm{NCT}\;\mathrm{in}\;\mathrm{film}}{\mathrm{Mass}\;\mathrm{of}\;\mathrm{NCT}\;\mathrm{in}\;\mathrm{feed}}\times 100\%$$

### 2.5. In Vitro NCT Release Study

NCT films were placed into a beaker containing 20 mL of water as a medium [9]. The beaker was placed over a magnetic stirrer, and the temperature of the assembly was maintained at 37 ± 2 °C throughout the experiment. During the experiment, rpm was maintained at 50 rpm. Samples (1 mL) were withdrawn at definite time intervals and replaced with equal amounts of fresh water. After 2 dilutions with ethanol, the samples were analyzed using HPLC following the method specified in Section 2.2.2.

Various kinetic models were used to describe the drug release kinetics and mechanism of drug release. The *in vitro* dissolution data were fitted to the zero-order model, First-order model, Higuchi matrix model, and Korsmeyer–Peppas empirical power law to obtain the best-fit model.

(a) The zero-order model explains the systems where drug rate release does not depend on its concentration. The release rate data were fitted into Equation (4), as follows:

(4) $$Q_{t}=Q_{0}+k_{o}\times t$$

where *Q*_t_ is the amount of drug dissolved in time (*t*), *Q_o_* is the initial amount of drug in the solution, and *k*_o_ is the zero-order release constant.

(b) The first-order model explains the release from the system where the drug release rate is concentration dependent. The first 60% of the release data points were fitted to Equation (5), as follows:

(5) $$Log\;Q_{0}\;-Log\;Q_{t}=\frac{k_{1}\;\times t\;}{2.303}$$

where *Q*_o_ is the initial concentration of the drug, *Q*_t_ is the amount of drug dissolved in time (*t*), and *k*_1_ is the first-order rate constant.

(c) The Higuchi matrix model describes the release of drugs from the insoluble matrix as a square root of the time-dependent process based on Fickian diffusion [19]. The release rate data were fitted to Equation (6), as follows:

(6) $$Q_{t}=k_{H}\times t^{1/2}$$

where *Q_t_* is the amount of drug released in time (*t*) and *k*_H_ is the Higuchi diffusion constant.

(d) The Korsmeyer–Peppas empirical power law describes the drug release from a polymeric system, as derived by Korsmeyer et al. [20]. The first 60% of the release data points were fitted to Equation (7), as follows:

(7) $$\frac{M_{t}}{M_{\infty }}=k\times t^{n}$$

where *M*_t_/*M*_∞_ is the fraction of drug released at time (*t*), *k* is the structural and geometrical constant, and n is the release exponent.

### 2.6. Statistical Analysis

The statistical evaluation of percentage extract yield and NCT content in tobacco leaves was performed using ANOVA; the characterization of NCT fast dissolving films was performed with the unpaired t-test using SPSS software version 17.0 (SPSS Inc., Chicago, IL, USA). The data are presented as mean ± SD, and *p* < 0.05 was considered to indicate significant differences.

## 3. Results and Discussion

### 3.1. Tobacco Leaf Extract and NCT Yield Percentage

**Figure 2:**
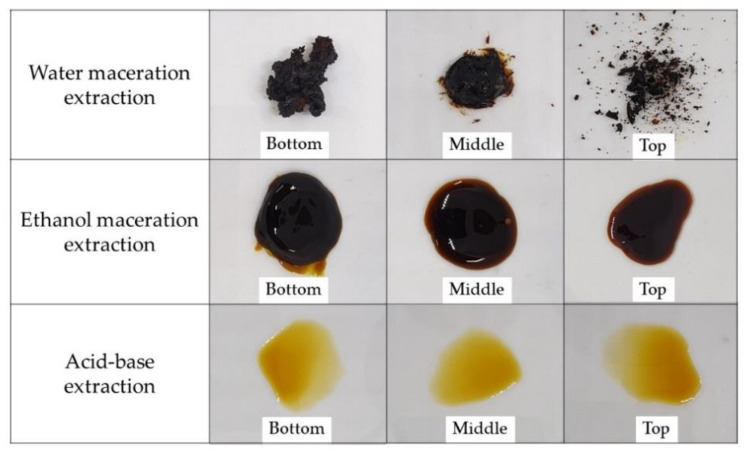
Tobacco leaf extract from different leaf positions using the maceration extraction method with water and ethanol as well as the acid-base extraction method.

The tobacco leaf extract from different leaf positions by maceration extraction with water and ethanol as well as acid-base extraction exhibited various appearances and viscosities (Figure 2). The most viscous extract was the maceration with water, which showed a solid to semisolid state. In comparison, ethanol extracts had a moderate viscosity that identified as a semisolid to a liquid state, whereas the extracts from acid-base extraction showed an oily liquid extract. The results of the percentage yield and NCT content from different extraction methods and parts of the tobacco leaves are shown in Table 1. It was found that the extraction technique affects percentage yield and NCT content. The acid-base extraction method presented the highest NCT content with the lowest yield of extract compared to the maceration extraction method from both solvents. When comparing within the maceration extraction method, the yield percentage of tobacco leaf aqueous extract showed a higher amount than the ethanolic extract. However, the NCT content obtained by ethanol maceration was greater than that of the aqueous solvent, which indicated the type of solvent also affected the NCT content. In 2001, Jones et al. compared several extraction methods and described that maceration with solvent extraction was a method that yielded favorable nicotine content and alkaloid tobaccos [21]. Similarly, Saengwan et al. (2016) extracted a similar cultivar of tobacco leaves to control chilli thrips in farmers’ fields using the maceration extraction method. They found that extraction with water for 24 h gave the highest amount of nicotine [22]. For the use of solvents, Puripattanavong et al. (2013) suggested that using methanol and ethanol gave the highest yield percentage—17.91–19.55%—compared with other solvents [23]. However, acid-base extraction is specific to the alkaloid group. In general, NCT is in a non-ionized form that is very soluble in organic solvents. Reaction with bases or acids leads to a salt form or ionized form, which increases water solubility. Tantullavetch et al. (2007) reported that nicotine extraction yield from tobacco leaves with acid-base extraction was approximately 4.2% [12], which is similar to the results of our present study. HPLC analysis suggested different parts of the tobacco leaves contained different amounts of NCT; the top part of the leaf had the highest NCT content. Other researchers have also reported a similar observation with other types of tobacco leaves, with the nicotine level increasing from bottom to top leaf positions of the tobacco plant [24,25,26]. Usually, all three parts of tobacco leaves are used for cigarette *manufacturing*. Proportional mixing of different types of tobacco leaves produces a variable taste of cigarettes. The top leaf position is also the most desirable by smokers as it has a more pleasant smell and taste. The literature reviews have reported that various chemical elements of each part of the tobacco leaf are of the same quality but have different quantities [27]. The extract from the top part of tobacco leaves using acid-base extraction yielded the highest amount of NCT and was therefore suitable for further study.

### 3.2. NCT Fast Dissolving Films

**Figure 3:**
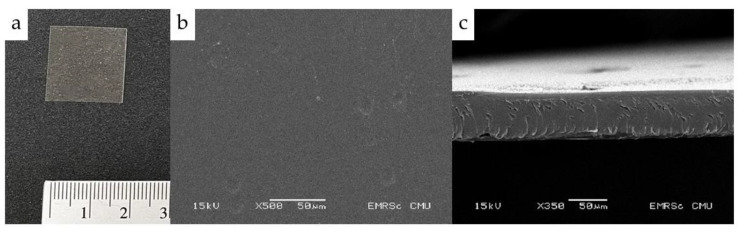
NCT fast dissolving film visual image (**a**); SEM images of the surface (**b**) and cross-section (**c**) at 500× and 350× magnification, respectively.

The casting of solution on a Petri dish enables high-quality results of film properties that can be replicated. The thickness of the film depends on the quantity of the solution poured out on the dish. The obtained NCT fast dissolving films by solvent casting technique exhibited a translucent light brown color due to the tobacco leaf extract. Scanning electron microscopy images of films are shown in Figure 3. The morphology of the NCT fast dissolving films appeared homogeneous and continuous, indicating the uniform distribution of NCT in the film formulation. The thicknesses of NCT fast dissolving films was approximately 70 µm.

The result of thickness, weight, and disintegration time of NCT fast dissolving films is shown in Table 2. The thickness of NCT film measured using a micrometer was in agreement with the one obtained from SEM (Figure 3c). There was a difference in the film weight and disintegration time between NCT films and blank films (*p* < 0.05). NCT increased the film weight, and the NCT film decomposed more slowly than the blank films. The results suggested that NCT might affect the polymeric film structure. Choi et al. (2016) reported that HPMC-containing essential oils exhibited strong physical and mechanical properties [28]. The disintegration time of the NCT fast dissolving films was less than 30 s, which is an acceptable disintegration time for fast dissolving film [29]. The results for mechanical properties and NCT loading efficiency are shown in Table 3. This result indicated that the tobacco leaf extract and NCT incorporated in the film had significantly increased tensile strength, percent elongation at break, and Young’s modulus values. This result was different from the oily drug-like essential oil incorporated into formulations, which usually influences the film’s mechanical properties by decreasing tensile strength and increasing the film’s brittleness [30,31,32]. NCT is soluble in water; therefore, it is miscible with HPMC solution. It did not negatively disrupt the polymer matrix’s continuity of HPMC, thus increasing film flexibility. NCT fast dissolving films exhibited very high NCT loading contents.

### 3.3. In Vitro NCT Release Study

Saliva is a liquid that contains more than 99% water, with trace amounts of ions and mucin [33]. The ions and mucin can reduce swelling and drug release capacity of anionic film polymer such as sodium alginate and other ion-containing element formulations. However, they did not affect nonionic polymers such as HPMC [13]. The mucin affected mucoadhesive property of HPMC polymer on the buccal epithelium [34]. Therefore, water was able used as a medium, without interfering with the drug release profile. Cilurzo et al. (2010) used a large amount of water (300 mL) as a medium [9], but the average volume of saliva in the oral cavity is approximately 20 mL/h during waking time. The stimulated flow rate was shown to range between 0–7 mL/min [33]. Thus, the medium volume (20 mL) and the renovation rate of the release medium (1.5 mL/min for first 60% of the drug release profile) of this study are suitable and resemble the oral cavity condition. Because of a low volume of the salivary fluid and its turnover rate, sink conditions could not completely occur in the buccal mucosa [35]. The sink conditions refer to a volume of medium that is at least three times larger than that needed to form a saturated solution of the drug substance. The overall concentration of the sample in the dissolution medium (φ) is less than one-third of the saturation solubility [36]. However, NCT is soluble in water at temperatures lower than 60.8 °C (1 × 10^6^ g/L) [37]; thus, the calculated φ value is 0.0001, which is less than one-third. Therefore, this *in vitro* release experiment was considered as a sink condition.

**Figure 4:**
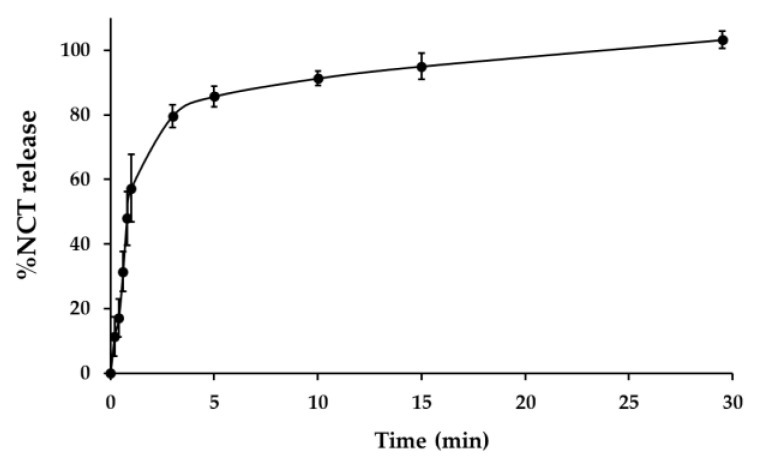
*In vitro* drug release profile of NCT fast dissolving film.

The drug release profile of NCT fast dissolving film is presented as a plot, being a relationship between NCT release percentage and time, as shown in Figure 4. The release percentage is defined as the ratio of drug released after the selected time period to the total amount of drug released. The drug release profile of NCT demonstrated a rapid initial release of 80% after the first 3 min of the experiment. The release of NCT at the end of the experiment (30 min) was 103.18 ± 2.71%. The release of NCT from fast dissolving film was assumed to be similar to that of cigarette smoking, which occurs for approximately 5 min. The previous study reported that NCT intake by cigarettes (1.1 mg/cigarette) and chewing gum (2 mg/piece) reached peak plasma drug concentrations for 5 and 30 min, respectively [38]. The slow absorption rate of NCT from chewing gum will not fulfill a smoker’s nicotine craving fast enough. However, it is necessary to perform a release experiment of NCT fast dissolving film and chewing gum under the same conditions in order to obtain comparable results. Chauhan and Madan (2012) prepared nicotine hydrogen tartrate loaded in fast dissolving films, using HPMC E3 and E5 as film polymers, and reported that nicotine hydrogen tartrate could release more than 70% in 5 min and approached plateau levels in less than 20 min [39]. This NCT release behavior is also similar to a previous report [40,41] which used HPMC as a film polymer, and can be considered as a fast dissolving film.

The kinetics of drug release can be determined by one or more mechanisms, depending on the composition of the matrix, the preparation method, the drug release dissolution medium, and biodegradation of polymeric chains [42,43,44]. In the present study, the release mechanism of NCT from NCT rapid dissolution film was evaluated using four mathematical models: the zero-order model, the first-order model, the Higuchi matrix model, and Korsmeyer–Peppas empirical power law, as presented in Table 4. According to each model, appropriate mathematical models can be selected based on the value of the correlation coefficient (*r*^2^) obtained from the linear regression analysis. The results indicated that the release behavior of NCT from NCT film was best fit to the Higuchi matrix model, suggesting that the drug release mechanism was diffusion from polymeric systems. Maheswari et al. (2014) formulated mouth dissolving films of amlodipine besylate using HPMC as a film polymer, similar to the present study. The developed film completely released within 60 s, with diffusion release kinetics [45]. There have been no previous studies reported on the NCT release kinetic from fast dissolving film. However, there was a study on the sustained release of NCT buccal film using HPMC and sodium alginate as film polymers, which showed prolonged drug release for 4 h. The NCT release data were fit to the Korsmeyer-Peppas model, with an n value of less than 0.24, which indicated the NCT released from the matrix by diffusion [46]. HPMC as a film-forming material is classified into several grades, depending on the degree of hydroxypropyl substitution and methoxy substitution. The hygroscopic nature of HPMC can increase the water uptake capability of the films and supports the Fickian diffusion mechanism.

## 4. Conclusions

The present study investigated the influence of extraction methods and the tobacco leaf positions that can provide the highest NCT content. The results demonstrated that the top-part tobacco leaf extract from the acid-base extraction method has the highest amount of NCT, although it yields only a small amount. NCT fast dissolving film containing 2 mg of NCT using HPMC E15 as a single polymer was successfully produced. The tobacco leaf extract and NCT highly influences the disintegration time and mechanical properties of film compared to blank film without drugs. The drug release profile demonstrated a rapid initial release of NCT within 3 min. The drug release kinetics can be explained by the Higuchi matrix model, which states that the drug release is controlled by the diffusion of a small liquid into a polymer matrix according to Fickian diffusion. The developed fast dissolving films can be modified as a suitable drug delivery system for other water-soluble medicines.

## Figures and Tables

**Table 1:** Yield extract percentage and nicotine (NCT) content from different extraction methods and parts of tobacco leaves.

Tobacco Leaf Extract		% Yield of Tobacco Leaf Extract	% Yield of NCT Content in the Extract
Water maceration extraction	Bottom	47.33 ± 1.21 ^a^	1.27 ± 0.12 ^f^
	Middle	42.39 ± 3.58 ^a^	5.72 ± 0.58 ^g^
	Top	47.61 ± 7.74 ^a^	12.07 ± 0.32 ^h^
Ethanol maceration extraction	Bottom	14.61 ± 0.07 ^b^	10.78 ± 0.45 ^h^
	Middle	14.95 ± 0.46 ^b^	15.31 ± 0.23 ^i^
	Top	24.71 ± 1.51 ^c^	16.99 ± 1.17 ^i^
Acid-base extraction	Bottom	3.14 ± 0.54 ^d^	43.28 ± 1.43 ^j^
	Middle	3.27 ± 0.66 ^d^	57.19 ± 1.46 ^k^
	Top	6.18 ± 1.30 ^e^	63.17 ± 1.10 ^m^

Note: For each test, means with the same letter are not significantly different. Thus, means with different letters, e.g., “a” and “b”, are statistically different (*p* < 0.05).

**Table 2:** Thickness, weight, and disintegration time of NCT fast dissolving films.

Film	Thickness (mm)	Weight (g)	Disintegration Time (s)	Normalized Disintegration Time (s)
NCT Film	0.070 ± 0.001 ^a^	0.0314 ± 0.0010 ^a^	19.95 ± 1.55 ^a^	19.96 ± 1.64 ^a^
Blank	0.079 ± 0.008 ^a^	0.0266 ± 0.0022 ^b^	11.63 ± 3.49 ^b^	10.42 ± 3.54 ^b^

Note: For each test, means with the same letter are not significantly different. Thus, means with different letters, e.g., “a” and “b”, are statistically different (*p* < 0.05).

**Table 3:** Mechanical properties and NCT loading efficiency of NCT fast dissolving films.

Film	Tensile Strength (N/mm^2^)	Elongation at Break (%)	Young’s Modulus (N/mm^2^)	NCT Loading Efficiency (%)
NCT Film	7.23 ± 0.31 ^a^	7.81 ± 0.08 ^a^	225.98 ± 7.38 ^a^	98.02 ± 6.12 ^a^
Blank	4.18 ± 0.07 ^b^	4.93 ± 0.25 ^b^	120.10 ± 3.57 ^b^	-

Note: For each test, means with the same letter are not significantly different. Thus, means with different letters, e.g., “a” and “b” are statistically different (*p* < 0.05).

**Table 4:** Release kinetic data of NCT film using various kinetic models.

Kinetic Model	Parameter	
Zero-order	*r* ^2^	0.5333
	*k*_0_ (min^−1^)	2.5057
First-order	*r* ^2^	0.5287
	*k*_1_ (min^−1^)	0.0136
Higuchi matrix	*r* ^2^	0.9794
	*k*_H_ (min^1/2^)	45.057
Korsmeyer–Peppas	*r* ^2^	0.9636
	*k* (min^−n^)	1.7434
	n	0.3472

## Data Availability

Data is contained within the research article.
